# Comparative analysis of rumen metagenome, metatranscriptome, fermentation and methane yield in cattle and buffaloes fed on the same diet

**DOI:** 10.3389/fmicb.2023.1266025

**Published:** 2023-11-09

**Authors:** Pradeep K. Malik, Shraddha Trivedi, Atul P. Kolte, Archit Mohapatra, Siddharth Biswas, Ashwin V. K. Bhattar, Raghavendra Bhatta, Habibar Rahman

**Affiliations:** ^1^ICAR-National Institute of Animal Nutrition and Physiology, Bangalore, India; ^2^International Livestock Research Institute, South Asia Regional Office, New Delhi, India

**Keywords:** buffaloes, cattle, metagenome, metatranscriptome, methane yield, rumen

## Abstract

A study to compare the rumen microbial community composition, functional potential of the microbiota, methane (CH_4_) yield, and rumen fermentation was conducted in adult male cattle and buffaloes fed on the same diet. A total of 41 phyla, 169 orders, 374 families, and 1,376 microbial genera were identified in the study. *Bacteroidetes* and *Firmicutes* were the two most dominant bacterial phyla in both cattle and buffaloes. However, there was no difference in the abundance of *Bacteroidetes* and *Firmicutes* in the rumen metagenome of cattle and buffaloes. Based on the abundance, the *Proteobacteria* was the 3rd largest phylum in the metagenome, constituting 18–20% in both host species. *Euryarchaeota* was the most abundant phylum of the methanogens, whereas *Methanobacteriales* and *Methanobrevibacter* were the most abundant orders and genera in both species. The methanogen abundances were not different between the two host species. Like the metagenome, the difference between the compositional and functional abundances (metagenome vs. metatranscriptome) of the *Bacteroidetes* and *Firmicutes* was not significant, whereas the proteobacteria were functionally less active than their metagenomic composition. Contrary to the metagenome, the *Euryarchaeota* was the 3rd most functional phylum in the rumen and constituted ~15% of the metatranscriptome. *Methanobacteriales* were the most functional methanogens, accounting for more than 2/3rd of the total archaeal functionality. These results indicated that the methanogens from *Euryarchaeota* were functionally more active as compared to their compositional abundance. The CH_4_ yield (g/kg DMI), CH_4_ emission (g/kg DDM), dry matter (DM) intake, and rumen fermentation did not vary between the two host species. Overall, the study established a substantial difference between the compositional abundances and metabolic functionality of the rumen microbiota; however, feeding cattle and buffaloes on the same diet resulted in similar microbiota composition, metabolic functionality, and CH_4_ yield. Further studies are warranted to investigate the effect of different diets and environments on the composition and metabolic functionality of the rumen microbiota.

## Introduction

Methane (CH_4_), with an average atmospheric concentration of 1,890 ppb (Dlugokencky, 2021), is the second most significant greenhouse gas in the atmosphere after carbon dioxide (EPA, 2023). The annual increase in atmospheric CH_4_ concentration is 10 parts per billion (Yusuf et al., 2012). Together, natural and anthropogenic sources emit 558 Tg (teragram) of CH_4_ per year, with 188 Tg coming from agricultural and waste-related human activities. After the removal of about 548 Tg of CH_4_ through various sinks, 10 Tg is annually added to the atmospheric CH_4_ pool. Livestock are held accountable for 15% of anthropogenic greenhouse gas emissions (Gerber et al., 2013), and enteric fermentation, with an annual emission of 87–97 Tg, remains one of the largest sources in the agriculture sector (Chang et al., 2019). Globally, cattle and buffaloes are two major emitters of enteric CH_4_ emissions and aggregately contribute 90% of total enteric CH_4_ (FAO, 2021). India possesses 192.5 and 110 million cattle and buffaloes (GOI, 2019), aggregately accountable for an annual enteric CH_4_ emission of 7.83 Tg (Bhatta et al., 2019).

Methanogenesis, due to the sizable loss of feed energy (Johnson and Johnson, 1995; Guan et al., 2006) is generally considered an inefficient process, but the involvement of methanogens in the removal of metabolic H_2_ via CH_4_ makes this process an obligation for the host animal rumen. Archaea are not as diverse as bacteria, but the distinct and specific substrate requirements make the community complex. Previous studies reported buffaloes have a different microbial profile and are better fiber digesters than cattle (Wanapat et al., 2000; Lapitan et al., 2008; Chanthakhoun et al., 2012; Wang et al., 2020). In a metagenomic study, Iqbal et al. (2018) concluded that cattle had a higher abundance of *Bacteroidetes and Prevotella* and a lower abundance of *Firmicutes* as compared to buffaloes fed a similar diet. They also reported a relatively higher abundance of *Methanobrevibacter* in cattle. On the contrary, Malik et al. (2021) recently compared the CH_4_ and rumen methanogens diversity between cattle and buffaloes fed on the same diet and concluded that the abundance of prominent methanogens was not different and host species had a limited influence on the archaeal community composition and CH_4_ yield. Similarly, Asai et al. (2021) also reported a similar density of cellulolytic bacteria between swamp buffalo and cattle fed on the same diet and concluded that the microbial community structure might be dependent on feed rather than animal species.

All these studies rely on the abundances of the rumen microbiota in different host species; however, it may not be necessary that a microbe with a prominent abundance also have proportional functional capabilities. Metatranscriptomics of the rumen microbiota may assist in understanding the functional potential better than metagenomics (Li et al., 2019). To the best of our knowledge, studies comparing the community composition with the microbial functional potential between cattle and buffaloes on the same diet and under the same environmental conditions are not available. Therefore, this study was undertaken to compare the rumen microbial community composition and functional potential of the microbiota between cattle and buffaloes fed on the same diet and the association between metagenomic composition and functional capabilities. The study also aims to investigate the impact of microbial communities and their functionality on CH_4_ emissions and rumen fermentation in cattle and buffaloes.

## Materials and methods

### Ethical approval

The experiment was carried out at the Livestock Experimental Unit of the ICAR-National Institute of Animal Nutrition and Physiology, Bangalore, India. The animal study, including the procedure for the handling of animals and the collection of gas and ruminal fluid samples, was approved by the Committee for Control and Supervision of Experiments on Animals (CPCSEA), Ministry of Fisheries, Animal Husbandry, and Dairying, Government of India (Approval no. NIANP/IAEC/1/2020/5).

### Animal feeding and management

Six male adult crossbred cattle (BW 500 ± 26.5 kg) and six male adult buffaloes (BW 261 ± 17.5 kg) were used to unravel the difference in rumen whole metagenome, transcriptome, CH_4_ yield, and fermentation pattern between the two host species fed on a similar diet. The animals of both species during the entire experiment were housed in the same environmental conditions to overrule the impact of climatic factors on the rumen microbiome composition and functions. The experimental animals were individually housed in tail-to-tail fashion in an east–west oriented cemented shed. The cemented walls of the shed on the north and south walls had wire fences above 1.8 m height. The shed had provisions for individual feeding and watering. Ten days before the commencement of feeding, the animals were dewormed with fenbendazole at 5 mg/kg BW. Animals of both species were fed *ad libitum* on a diet comprising finger millet straw (*Eleusine coracana*) and para (*Brachiaria mutica*) grass in a 70:30 ratio. The feed was offered daily at the 9:00 h, while clean drinking water was accessible to the animals throughout the 24 h. The experiment was conducted for 45 days, including 30 days of preliminary feeding followed by 15 days of enteric CH_4_ measurement and digestibility trials.

### Rumen fluid collection

On the last day of the experiment, the rumen fluid samples (45 mL) were collected from the individual animals at 3 h post-feeding using a nylon stomach tube (length 2 m) connected to a vacuum pump (Mityvac 8000, Lincoln Industrial, St. Louis, United States) and an airtight collection vessel (Malik et al., 2015b; Thirumalaisamy et al., 2022a,b). The first 30 mL of rumen digesta sample to avoid saliva contamination was thrown away, and the subsequent 45 mL of digesta was retained for different purpose. The rumen fluid was split into three 15-ml subsets that were used to isolate nucleic acids, estimate ammonia-VFA, and enumerate protozoa. The subsets for the nucleic acid analysis and the ammonia/VFA analysis were put in an ice box and taken to the lab. The third subset, which was used to count the number of protozoa, was taken to the lab without being put on ice. For nucleic acid isolation, the rumen digesta containing both the fluid and solid fractions was used without filtration, whereas the supernatant obtained after the initial centrifugation at 11300 × g at 4°C for 15 min was preserved for the ammonia-VFA estimation. The supernatant was preserved for the ammonia-N estimation after 2-3 drops of saturated HgCl_2_, whereas the metaphosphoric acid (25%) in 1:4 (v/v) was added to the supernatant preserved for the VFA estimation. The third subset of the digesta sample was used for the protozoal enumeration on the same day.

### Nucleic acids extraction

#### DNA isolation

The RBB + C method of Yu and Morrison (2004) was used to isolate gDNA from the digesta samples collected from cattle and buffaloes. 0.25 g sample containing both solid and liquid fractions was dissolved in one ml of lysis buffer and transferred to a two-ml sterile screw cap tube (BioSpec, United States) containing 0.5 g of 0.1 mm sterilized zirconia beads (BioSpec, United States). The content was homogenized for 3 min at the maximum speed using a mini bead beater (Biospec, USA) and then incubated at 70°C for 15 min with intermittent shaking. The supernatant was transferred to a two ml Eppendorf tube after centrifugation at 13,000× g. The bead beating was repeated with the remaining content in the screw cap tube after adding 300 μL of lysis buffer, and the supernatant was pooled. To precipitate the proteins and polysaccharides, 260 μL of 10 M ammonium acetate was added to the lysate tube and incubated on ice for 5 min. The content was centrifuged at 4°C for 10 min at 13,000× g. The supernatant was transferred to another *Eppendorf* tube, and an equal volume of isopropanol was added before mixing and incubation on ice for 30 min. After the incubation, the content was centrifuged at 4°C for 10 min at 13,000× g and the pellet, after removing the supernatant, was washed with 70% ethanol. The nucleic acid pellet was dissolved in 100 μL Tris-EDTA buffer. To remove RNA and protein, 2 μL DNase-free RNase (10 mg/mL) was added and incubated for 15 min at 37°C, followed by the addition of 15 μL of proteinase K and 200 μL of AL buffer (Qiagen, Germany), and incubation at 70°C for 15 min. After adding 200 μL absolute ethanol and mixing the contents, the subsequent steps were performed as per the manufacturer’s instructions using the QIAamp DNA mini kit (Qiagen, Germany). The quality of the genomic DNA was checked with 0.8% agarose gel electrophoresis, while the DNA was quantified using Qubit 4.0 (Invitrogen, United States).

#### RNA isolation

For RNA isolation, the rumen digesta of the same batch was pelleted at low speed (10,000× g) for 5 min, resuspended in 100 μL RNAlater stabilizing solution (GCC Biotech, India), and preserved until processing (Comtet-Marre et al., 2017). The RNA was extracted from a 0.25 g preserved digesta sample using the RNeasy^®^ PowerMicrobiome^®^ Kit (Qiagen) as per the manufacturer’s instructions. The RNA purity and concentration were checked by a Qubit 4.0 fluorometer (Invitrogen, United States) using the Qubit RNA BR Assay Kit (ThermoFisher Scientific, United States).

### Rumen metagenome sequencing

The DNA samples were sent to an external sequencing facility (Clevergene Biocorp, Bangalore, India) for sequencing on the HiSeq2500 (Illumina Inc., United States) platform. Metagenomic libraries were prepared using the NEBNext^®^ Ultra^™^ II FS DNA Library Prep Kit for Illumina^®^ (New England Biolabs). Initially for fragmentation, 100–500 ng of gDNA were fragmented to 350 bp at 37°C for 15–20 min using NEBNext Ultra II FS Reaction Buffer and Ultra II FS Enzyme Mix in a PCR thermal cycler with the following incubation steps: 30 min at 37°C, 30 min at 65°C and hold at 4°C. For adaptor ligation, 35 μL of fragmented DNA was mixed with NEBNext Ultra II Ligation Master Mix and NEBNext Adaptor for Illumina and incubated at 20°C for 15 min. According to the manufacturer’s instructions, the adaptor ligated DNA was size-selected with NEBNext Sample Purification Beads, and seven-cycle PCR amplification was performed with index primers (i5 and i7) and the following PCR conditions: initial denaturation at 98°C for 30 s, denaturation at 98°C for 10 s, annealing at 65°C for 75 s, and final extension at 65°C for 5 min. The validation of PCR-enriched libraries was done using the Agilent D1000 ScreenTape System in a 4150 TapeStation (Agilent Technologies, Germany). The libraries were loaded onto HiSeq2500 for cluster generation and sequencing, and 150 bp paired-end reads (2 × 150 bp) were obtained.

### Metatranscriptome sequencing

The RNA samples were sent for transcriptome sequencing on the HiSeq2500 platform (Illumina Inc., United States) to an external facility (Clevergene Biocorp, Bangalore, India). The RNA quality was ascertained by the TapeStation System (Agilent Technologies), and samples with ≥7.0 RNA Integrity Number were used for generating the metatranscriptome libraries. The rRNA was depleted by FastSelect hybridization followed by bead clean-up using the QIAseq FastSelect −5S/16S/23S Kit (Qiagen, United States). The metatranscriptomic libraries were prepared using the NEBNext Ultra II RNA Library Prep Kit (New England Biolabs, United States). The depleted RNA was chemically fragmented in a magnesium-based buffer at 94°C for 10 min and primed with random hexamers. The synthesis of the first and second strand cDNA from the primed RNA was done using NEBNext First Strand Synthesis Enzyme Mix and NEBNext Second Strand Synthesis Enzyme Mix, respectively. The double-stranded cDNA was then purified using NEBNext Sample Purification Beads and fragments were subjected to end repair as well as loop adapter ligation. The 400–600 bp-sized products were selected using NEBNext Sample Purification Beads and enriched by 12 cycles of PCR using the NEBNext Ultra II Q5 master mix and NEBNext^®^ Multiplex Oligos for Illumina. The amplified products were purified using 0.9X AMPure XP beads (Beckman Coulter), and the library concentration was determined on a Qubit 3.0 Fluorometer (ThermoFisher Scientific, United States). The libraries were pooled and loaded onto the sequencer for cluster generation and paired-end sequencing (2 × 150 bp).

### Bioinformatics analysis

The quality of raw reads and adaptor contamination in the rumen metagenome and metatranscriptome were checked with FastQC v0.11.9 (Andrews, 2010). The adapters, low quality bases of Q < 30 and short reads of <100 bp, were removed using trimmomatic v0.39 (Bolger et al., 2014) with the following parameters: ILLUMINACLIP:TruSeq3-PE-2.fa:2:30:10 SLIDINGWINDOW:15:30 MINLEN:100 TRAILING:30 AVGQUAL:30. Further, the host sequence contamination in the metagenome and metatranscriptome trimmed reads was removed by BowTie2 v2.5.0 (Langmead and Salzberg, 2012) using reference host genome assemblies ARS-UCD1.2 (RefSeq assembly accession: GCF_002263795.1) for cattle and NDDB_SH_1 (RefSeq assembly accession: GCF_019923935.1) for buffaloes. The unmapped reads were saved using --un-conc for paired end data and --un for single end data. This unmapped data was considered clean reads, free from host contamination. The clean reads were taxonomically classified by Kraken2 (Wood et al., 2019), and the full report output was parsed in Pavian (v1.2.0, Breitwieser and Salzberg, 2020). The species richness (alpha diversity) in both metagenome and metatranscriptome samples was calculated at the genus level by the Shannon index in MicrobiomeAnalyst (Chong et al., 2020). To compare the diversity between cattle and buffaloes rumen metagenome and metatranscriptome, the beta diversity metrices was calculated using Bray–Curtis dissimilarity following the ordination method of Principal Coordinate Analysis (PCoA) in MicrobiomeAnalyst. The data at different taxonomic levels was scaled using total- sum scaling feature in MicrobiomeAnalyst, which divided the feature read counts clustered within the same OTU by the total number of reads in each sample (Paulson et al., 2013). The comparison of metagenome and metatranscriptome at different taxonomic levels between the two host species were performed using Wilcoxon rank sum test with Benjamini-Hochberg correction (FDR) for the adjusted *p* value of < 0.05.

### Comparison of compositional and functional abundances

To compare the differential abundance between the compositional (metagenome) and functional (metatranscriptome) microbiota at different taxonomic ranks, the output of Kraken2 was parsed in Pavian and analyzed in MicrobiomeAnalyst using DeSeq2 (Love et al., 2014). The compositional vs. functional microbiota at various taxonomic ranks was visualized using Sankey diagrams (v. 1.2.0, Breitwieser and Salzberg, 2020).

### Gene prediction and carbohydrate-active enzymes annotation

The paired-end and single-end (forward or reverse surviving) clean reads obtained from trimmomatic were assembled using MEGAHIT v1.2.9 (Li et al., 2015) with the default parameters and a minimum contig length of 1,000 bp. The assembled reads were used for prokaryotic gene prediction by MetaGeneMark (Zhu et al., 2010) with default parameters and the gene prediction algorithm GeneMark.hmm prokaryotic (version 3.25). The redundancy in the predicted proteins was removed using CD-Hit v4.8.1 clustering at a sequence identity threshold of 95% (Li and Godzik, 2006). The predicted genes were scanned for the candidate Carbohydrate-Active Enzymes (CAZymes) using DIAMOND (v2.0.15.153), HMMER v3.2.1 with a default cutoff *E*-value, and eCAMI using the standalone version of the dbCAN annotation tool for automated CAZyme annotation (Zhang et al., 2018). The output files generated by DIAMOND, HMMER, and eCAMI were combined, and hits reported in either of the above two tools were summarized. CAZymes encoding contigs were analyzed for different classes: auxiliary activities (AA), carbohydrate-binding modules (CBM), carbohydrate esterases (CE), glycoside hydrolases (GH), glycosyl transferases (GT), and polysaccharide lyases (PL).

### Enteric CH_4_ emission

After 30 days of feeding, the daily enteric CH_4_ emission in cattle and buffaloes was quantified employing the sulfur hexafluoride (SF_6_) tracer technique of Berndt et al. (2014). Brass permeation tubes, 34 mm long, 8.5 mm dia., with a 30 mm-deep, 4.8-mm-blind hole, served as the source of SF_6_. A 0.24-mm Teflon septum supported by a 2 μm-pore size SS frit was placed in the nut to control the release of gas from the brass tubes. The permeation tubes were charged with SF_6_ (805 ± 8.78 mg) in liquid nitrogen. The tubes were calibrated for SF_6_ release over 70 days by placing them at 39°C and recording the tubes’ weight weekly. The mean SF_6_ release rates (mg/d) from the brass permeation tubes were 3.93 ± 0.107. On the calibration of the release rate, the tubes were inserted into the cattle and buffaloes’ rumen 10 days prior to the commencement of the CH_4_ measurement study. The halters were assembled using nylon tube, capillary tube (Supelco, 56712-U, ID 1/16) and quick connectors (Swagelok, B-QC4-D-200) as per Williams et al. (2014). The PVC canister for the gas sampling in the background air was hung daily on the ventilated iron wire mesh fixed in the cement wall in the north direction of the shed. Throughout the CH_4_ measurement trial, the canisters were tied and removed from the animals at a fixed and consistent time every day. The initial and final pressures of the vacuumized and gassed PVC canisters were measured with a digital pressure meter (Leo 2, Keller). High-purity N_2_ gas was used for the dilution (2.50–3.50 folds) of breath and background samples for easy successive sub-sampling. The diluted gas samples were injected into the gas chromatograph (GC 2010 plus, Shimadzu, Japan) equipped with a flame ionization detector (FID) and an electron capture detector (ECD), for the estimation of CH_4_ and SF_6_ gasses, respectively. The GC conditions described previously by Malik et al. (2021, 2022a) and Thirumalaisamy et al. (2022b) were upheld for the estimation of gasses in the breath samples. The daily enteric CH_4_ emissions was calculated using the equation of Moate et al. (2014). A minimum of six successful collections of breath samples from the individual animals were ensured during the trial. The CH_4_ yield from cattle and buffalo fed a similar diet was calculated by taking into account the daily dry matter (DM) intake and enteric CH_4_ emission from each animal. The daily CH_4_ emission (g/d) was divided with the total DM intake to estimate the CH_4_ yield (g) on a uniform DM intake basis (per kg DMI). Similarly, the dry matter digestibility (DMD) was taken into consideration for calculating the CH_4_ emission (g) per unit of digestible dry matter (DDM) intake in both cattle and buffaloes.

### Protozoal enumeration

The rumen fluid collected from the cattle and buffaloes for protozoal enumeration was transported to the laboratory without being placed in an ice tray and processed immediately by mixing 1 volume of rumen fluid with 1 volume of formal saline. The sample was left at room temperature overnight. Protozoa enumeration was carried out using a phase-contrast microscope (Nikon Eclipse, Japan) at 10× objective in accordance with Kamra and Agarwal (2003). The protozoal numbers were expressed as ×10^7^ cells/mL. However, the morphological characterization of the protozoa was done as per Hungate (1966).

### Nutrient intake and digestibility

Concurrent with the CH_4_ measurement trial, the nutrient intake and digestibility study was also conducted in both species. Daily feed offered, refusals, and fecal output were weighed quantitatively for the individual animal, and representative samples were collected and dried at 80°C for 24 h. The nutrient intake (kg/d) was determined by considering the daily allowance and refusals, whereas the apparent digestibility (%) was determined by taking the weight of feed offered, refusals, and dung voided into consideration as per the equation given below. The total weight of dung voided by the individual animal over a period of 24 h was quantitatively collected in a plastic bucket covered with the appropriate lid, and an aliquot equivalent to 1/100th was taken for the DM estimation in a hot air oven at 100°C. Another aliquot of dung equivalent to 1/1,000th of the dung voided was preserved in 25% sulfuric acid for the ammonia estimation. The chemical constituents in dried and ground samples of feed, refusals, and feces were analyzed following the standard procedures. The crude protein (CP = N × 6.25) and ash content were analyzed in accordance with AOAC (2012), whereas the fiber constituents neutral detergent fiber (NDF) and acid detergent fiber (ADF) were determined according to Van Soest et al. (1991).

$\mathrm{Digestibility}\;\mathrm{coefficient}=\frac{\mathrm{Nutrient}\;\mathrm{intake}-\mathrm{Excretion}\;\mathrm{of}\;\mathrm{nutrient}}{\mathrm{Intake}\;\mathrm{of}\;\mathrm{nutrient}\;}$

### VFA and ammonia

The initially processed and preserved rumen fluid samples were thawed at room temperature for the estimation of VFA and ammonia nitrogen. The concentration of individual VFA was estimated as per Filípek and Dvořák (2009) using a gas chromatograph (Agilent 7890B, Santa Clara, United States) with the following conditions: temperature program: 59°C–250°C (20°C/min, 10 min), injector temperature: 230°C, and detector temperature: 280°C (Malik et al., 2021; Thirumalaisamy et al., 2022b).

The ammonia nitrogen was determined according to Conway (1957). One ml of boric acid was pipetted into the inner chamber, and an equivalent volume of sodium carbonate was pipetted into the outer chamber of the disk. In the outer chamber, 1 mL of ruminal fluid was pipetted opposite the sodium carbonate, and the disk was covered with the lid and left undisturbed at room temperature for 2 h. Thereafter, the mixed content was titrated against the 0.01 N sulfuric acid, and the ammonia concentration was determined.

### Statistical analysis

The data distribution was checked for normality (gaussian) using the built-in Shapiro–Wilk method in GraphPad Prism version 9.0. All the animal trait data were analyzed in GraphPad Prism version 9.0 using an unpaired *t* test, and based on the significance level of 0.05, the superscripts were placed wherever the differences between the two means for a given parameter proved significant.

## Results

### Alpha and beta diversity

**Figure 1:**
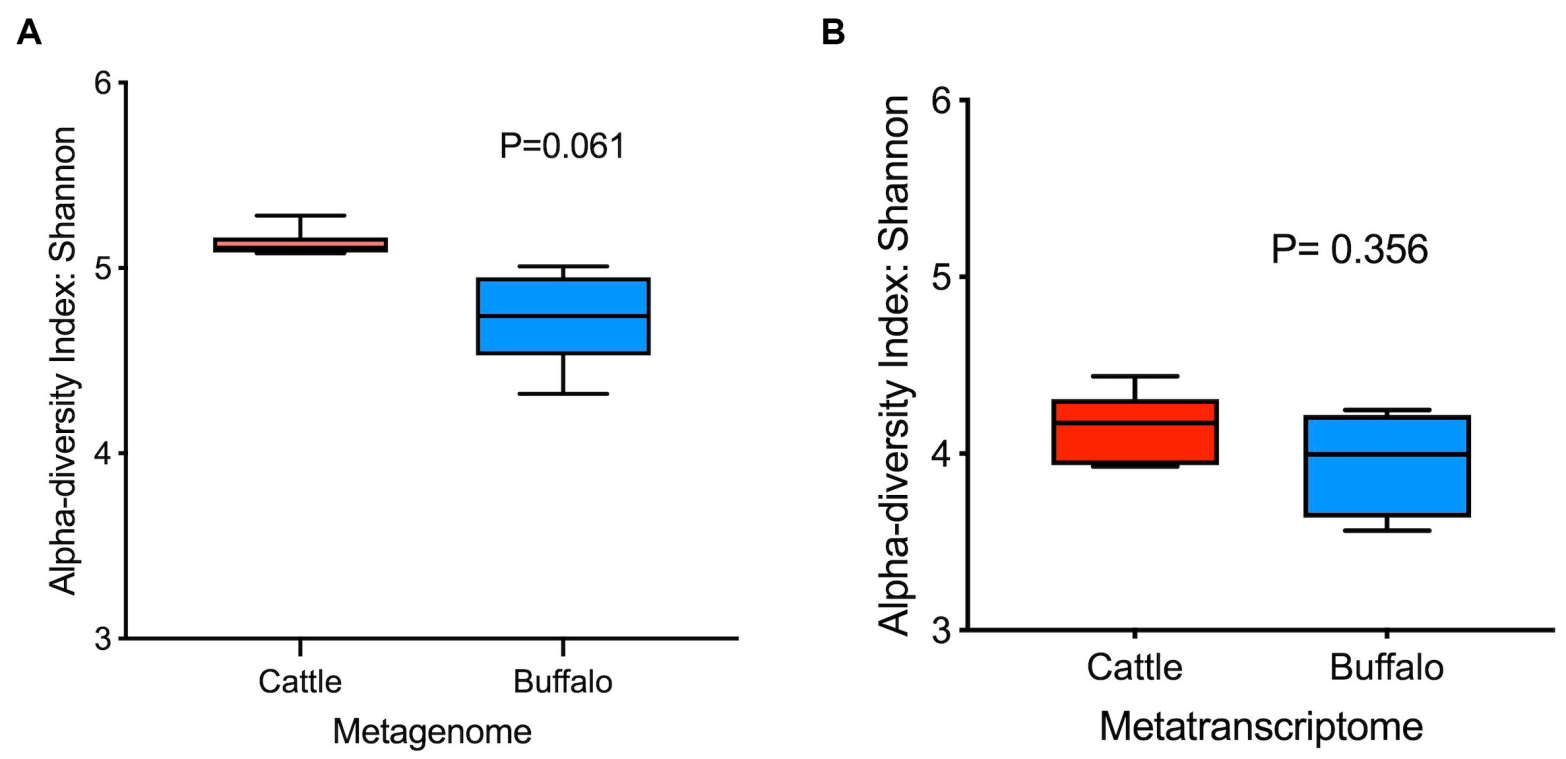
Alpha diversity based on the Shannon indices of **(A)** rumen metagenome (*N* = 6, 6) and **(B)** metatranscriptome (*N* = 5, 4). Values in parenthesis represent the number of metagenome/metatranscriptome samples in cattle and buffaloes, respectively.

**Figure 2:**
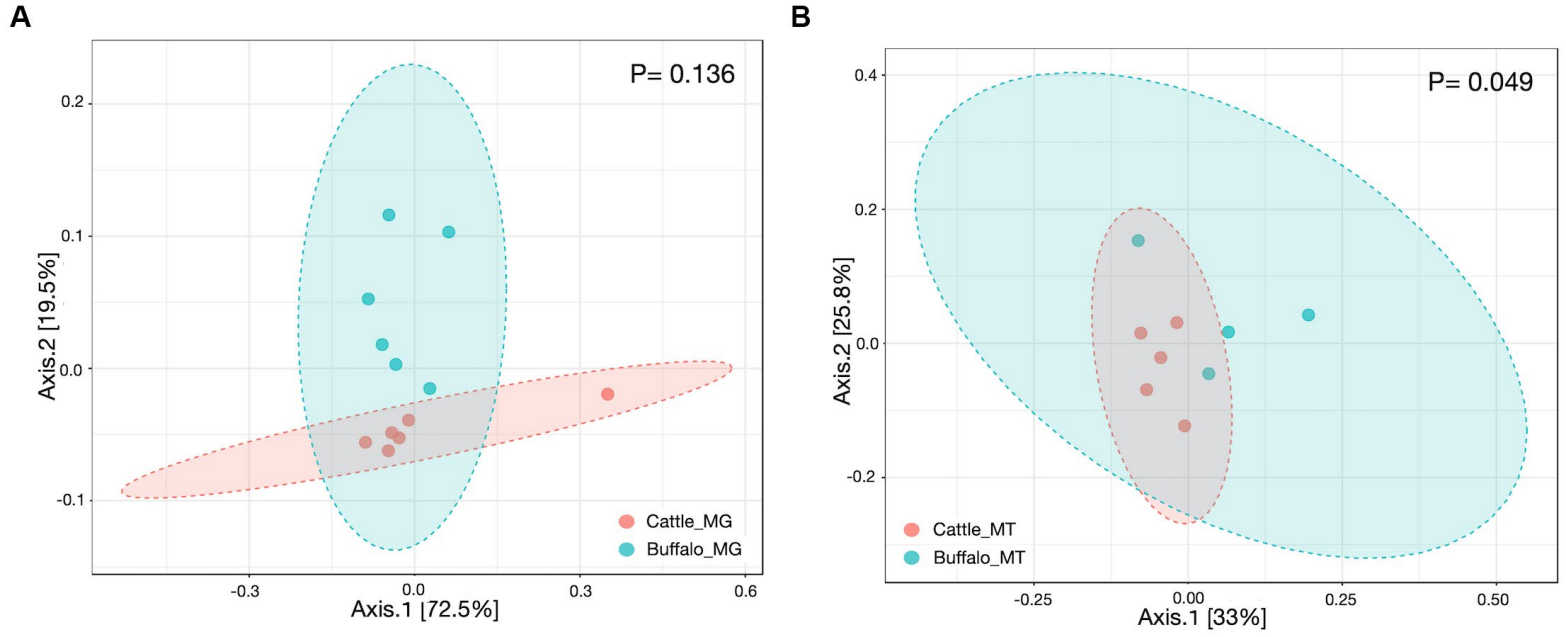
Beta diversity based on Bray-Curtis indices of **(A)** rumen metagenome (*N* = 6, 6) and **(B)** metatranscriptome (*N* = 5, 4). Values in parenthesis represent the number of metagenome/metatranscriptome samples in cattle and buffaloes, respectively.

The plateau of the rarefaction curves for both the metagenome and metatranscriptome samples (Supplementary Figure S1) demonstrated that the depth of sequencing was sufficient to cover the rumen microbial diversity. The Goods coverage index for both the metagenome and transcriptome was 1, which indicates that the generated reads sufficiently covered the microbial diversity in the rumen of cattle and buffaloes. The Shannon index was not significantly different (Figures 1A,B) for the metagenome (*p* = 0.061) and metatranscriptome (*p* = 0.356). In this study, the beta diversity assessed by Bray-Curtis was also non-significant for both the metagenome (*p* = 0.136) and the metatranscriptome (*p* = 0.049) (Figures 2A,B).

### Compositional abundance

A total of 202 million reads, with an average of 16.86 million reads per sample, were generated (Supplementary file S1). The total raw reads generated from the ruminal fluid samples of cattle and buffaloes were 97.9 and 104.3 million, respectively, whereas the mean of raw reads were 16.32 and 17.39 million in cattle and buffaloes, respectively. After trimmomatic quality filtration, a total of 8.7 million reads, including 4.2 million in cattle and 4.61 million in buffaloes, were removed. Further, due to host contamination, 0.70 and 0.17% reads were removed in cattle and buffaloes, respectively (Supplementary file S1). Finally, 58 GB of data with an average of 4.83 GB per sample were processed for taxonomic classification in Kraken2 and subsequently in MicrobiomeAnalyst.

**Figure 3:**
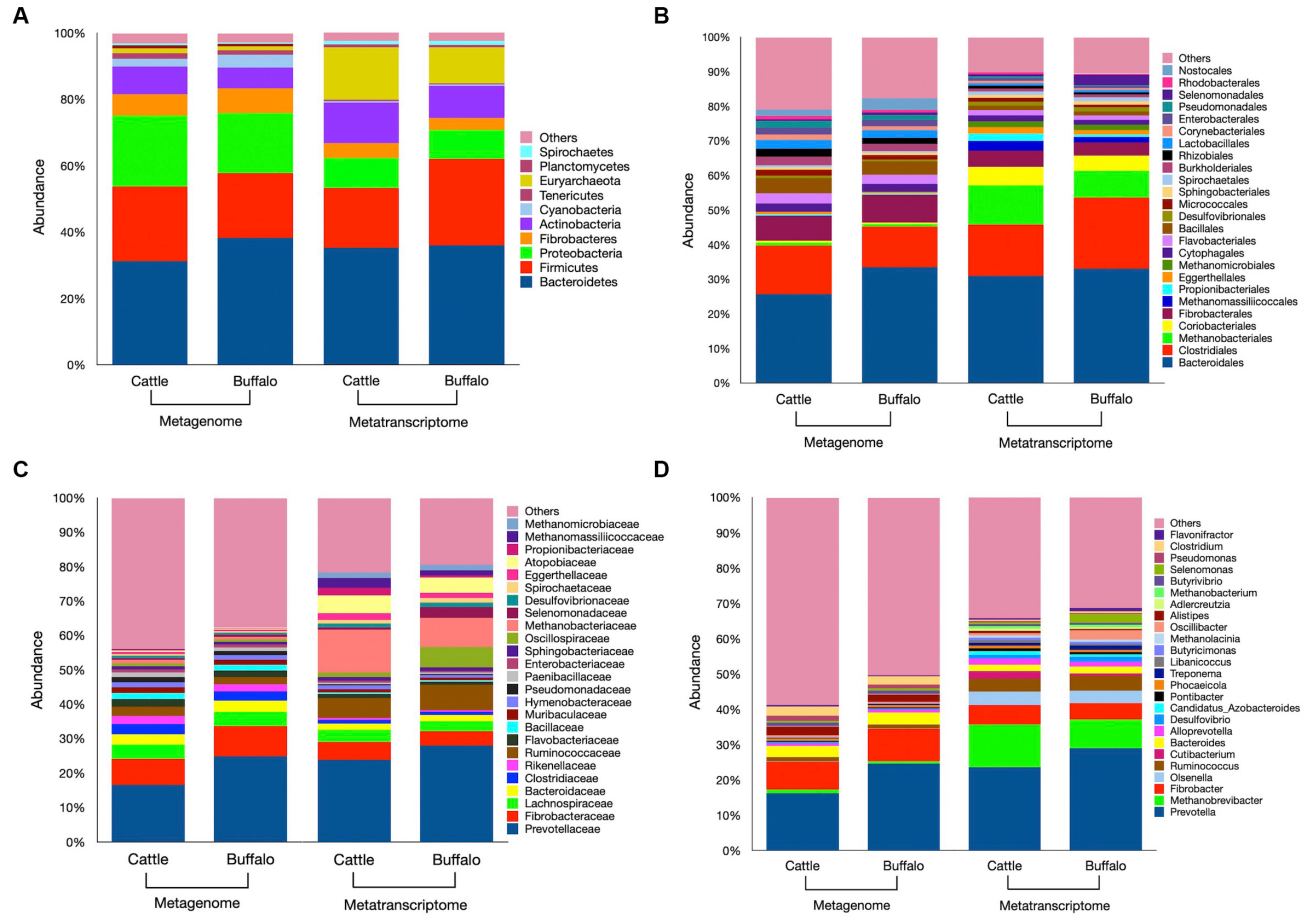
Metagenome and metatranscriptome profiles of rumen microbiota in cattle and buffaloes fed on a finger millet straw and para grass-based diet. Relative abundances of microbiota at the phylum level (**A**, top 10), order level (**B**, top 25), family level (**C**, top 25) and genus level (**D**, top 25) are depicted in bar plots. The “Others” category in the various bar plots represent, taxa other than the top categories specified above. Stacked bar plots prepared using mean values obtained from *N* = 6 metagenome samples in each species and *N* = 5 & 4 metatranscriptome samples in cattle and buffaloes, respectively.

In our study, a total of 41 phyla, 169 orders, 374 families, and 1,376 microbial genera were identified (Supplementary file S1). The top 10 phyla and top 25 orders, families and genera each based on their abundance are depicted in Figures 3A–D. The *Bacteroidetes* and *Firmicutes* were the two most dominant bacterial phyla in both cattle and buffaloes metagenome. These bacterial phyla aggregately constituted 53–58% of the total metagenome. The difference in *Bacteroidetes* (*p* = 0.35) and *Firmicutes* (*p* = 0.41) distribution between the two host species was not significant. The abundance of *Bacteroidetes* was negatively correlated with the abundance of *Firmicutes* in the metagenome (*r* = −0.81). The *Firmicutes* (F) to *Bacteroidetes* (B) ratio in the cattle and buffaloes rumen metagenome was 0.77 and 0.54, respectively (Supplementary file S1). The difference in *F/B* ratio between the two host species was significant (*p* = 0.042). The *Proteobacteria* was the 3rd largest phylum in the metagenome, constituted 18–20% in both host species. Similar to the *Bacteroidetes* and *Firmicutes*, the abundance of *Proteobacteria* between the cattle and buffaloes was also similar (*p* = 0.177). At the order level, the *Bacteroidales* constituted the largest fraction of the metagenome in both species (Figure 3B). However, their distribution between cattle and buffaloes was not different (*p* = 0.342). The next two dominant bacterial orders were *Clostridiales* (*p* = 0.342) and *Fibrobacterales* (*p* = 0.461) and their abundances were similar between the host species (Supplementary file S1). At the genus level, the *Prevotella* constituted 16–24% of the total metagenome, whereas the *Fibrobacter* and *Bacteroides* were the next two dominant bacterial genera. However, their distribution was also not different between cattle and buffaloes (Figure 3D).

*Euryarchaeota* was the most dominant phylum of methanogens in the rumen metagenome of cattle and buffaloes. Overall, the *Euryarchaeota* constituted the 8th most abundant phyla in the metagenome. The average distribution of the *Euryarchaeota* in the rumen metagenome of cattle and buffaloes was 1.48 and 1.10%, respectively (Figure 3A). *Methanobacteriales* were the most abundant methanogens in the rumen metagenome at the order level, constituted 0.95 and 0.67% of the metagenome in cattle and buffaloes. However, their abundance was not significantly different (*p* = 0.46) between the hosts. At the genus level, *Methanobrevibacter* constituted 1.01 and 0.70% of the total rumen metagenome in cattle and buffaloes and found to be the largest genus of methanogens with a comparable distribution (*p* = 0.759). *Methanobacterium* was another dominant genus of the methanogens and overall constituted the 24th dominant genus in the metagenome. Though other methanogens such as *Methanococcales, Methanomassiliicoccales*, *Methanomicrobiales*, *Methanosarcinales*, *Methylococcales*, and *Thermoplasmatales* were detected in the rumen, but at a very low frequency. These genera aggregately constituted 0.37–0.50% of the metagenome and their abundance was comparable between the host species (Supplementary file S1). Apart from the *Euryarchaeota*, the methanogens affiliated to others phylum such as *Crenarchaeota* and *Thaumarchaeota* were also detected in the rumen metagenome of cattle and buffaloes. However, their pooled abundance was ≤0.10% of the metagenome in both the hosts (Supplementary file S1). At an extremely low frequency (<0.0005%), the archaea belonging to the phylum *Candidatus Geothermarchaeota* was also identified in the metagenome.

### Functional abundance

A total of 232.67 million with an average of 19.4 raw reads per sample were generated from the rumen metatranscriptome of cattle and buffaloes in this study (Supplementary file S1). In cattle, a total of 117.9 with an average of 19.65 million raw reads per sample were generated, whereas the corresponding figures for total and average raw reads in buffaloes rumen metatranscriptome were 114.7 and 19.2 million, respectively. After trimmomatic quality filtration, a total of 4.74% of reads from the metatranscriptome were dropped. Moreover, 4.24 and 2.23% of the generated data were removed due to the host contamination in cattle and buffaloes, respectively. For taxonomic classification at various ranks, 66 GB of data were processed further in Kraken2 and subsequently in MicrobiomeAnalyst.

Similar to the metagenome, the metatranscriptomic analysis of the rumen microbiota revealed that *Bacteroidetes* and *Firmicutes* were the most common phyla in the rumen (Figure 3A), aggregately constituted 53 and 61.8% of the rumen metatranscriptome in cattle and buffaloes, respectively (Supplementary file S1). However, their distribution between cattle and buffaloes was comparable. The abundance of *Bacteroidetes* was negatively correlated with the abundance of *Firmicutes* in the metatranscriptome (*r* = −0.69). The *F/B* ratio in the rumen metatranscriptome of cattle and buffaloes was 0.52 and 0.81, respectively, and the difference between cattle and buffaloes rumen metatranscriptomes was not significant (*p* = 0.208). Similar to the metagenome, *Bacteroidales*, *Clostridiales,* and *Fibrobcterales* were three prominent orders of the bacteria and combinedly constituted 57 and 61% of the rumen metatranscriptome in cattle and buffaloes, respectively (Figure 3B). The abundances of the bacteria affiliated to these three dominant orders were similar between the two host species (Supplementary file S1).

Contrary to the metagenome, *Euryarchaeota* was 3rd largest phylum in the rumen, constituted 15.7 and 10.8% of the rumen metatranscriptome in cattle and buffaloes, respectively. Similar to the metagenome, the abundance of *Euryarchaeota* in the rumen metatranscriptome between cattle and buffaloes was not different (*p* = 0.75). Methanogens affiliated to the phylum, i.e., *Crenarchaeota* and *Thaumarchaeota* were also equally distributed in the rumen metatranscriptome of cattle and buffaloes. Among the archaea, the *Methanobacteriales* with a similar distribution between cattle and buffaloes (*p* = 0.69) were largely distributed in both the species and constituted 11.3 and 7.81% of the rumen metatranscriptome in cattle and buffaloes, respectively. After *Methanobacteriales*, *Methanomassiliicoccales* > *Methanomicrobiales* > *Methanosarcinales* > *Methylococcales* were the next most abundant methanogens in the rumen metatranscriptomes of two hosts. However, the distribution of all these methanogens at the order level was also comparable between the rumen metatranscriptomes of cattle and buffaloes (Supplementary file S1). *Methanobrevibacter* was the most prevalent genus, comprising 12 and 8% of the rumen Metatranscriptome in cattle and buffaloes, respectively (Figure 3D). As with higher ranks, the abundance of *Methanobrevibacter* did not differ between the metatranscriptomes of two hosts (*p* = 0.83).

### Compositional vs. functional abundance

*Bacteroidetes* and *Firmicutes* were two dominating phyla in both the metagenome and metatranscriptome of cattle and buffaloes (Supplementary file S1). Irrespective of host species, the pooled F/B ratio was 0.66 and 0.65 in the rumen metagenome and metatranscriptome, respectively, and the difference between the metagenome and metatranscriptome representing the compositional and functional abundances was not significant (*p* = 0.947). However, the *Proteobacteria* abundance in the rumen metatranscriptome was significantly lower (*p* < 0.0001) than the metagenome (Supplementary file S1). The comparison of compositional vs. functional abundance revealed a substantial decrease in the functionality of *Proteobacteria* in most of the orders affiliated to it. On the contrary, there were few orders where the functional abundance was relatively greater in the metatranscriptome (Supplementary file S1). More specifically, the bacteria affiliated to *Alteromonadales*, *Burkholderiales*, *Campylobacterales*, *Enterobacterales*, *Pasteurellales*, *Pseudomonadales*, and *Rhizobiales*, were functionally less active than their compositional abundances in the metagenome. On the other hand, the functional abundance of *Cardiobacteriales*, *Desulfovibrionales*, *Hydrogenophilales*, *Mariprofundales*, and *Syntrophobacterales* was significantly greater than their compositional abundance.

Contrary to the *Proteobacteria,* the overall functional abundance of *Actinobacteria* was higher (*p* = 0.001) than their compositional abundance (Supplementary file S1). An insight analysis of the phylum *Actinobacteria* at the order level revealed that the abundances of orders such as *Corynebacteriales*, *Streptomycetales*, *Bifidobacteriales,* and *Micrococcales* were significantly lower in the metatranscriptome as compared to the metagenome. On the other hand, functional abundance over the compositional abundances of *Coriobacteriales*, *Eggerthellales*, and *Propionibacteriales* was greater. Further, the data also indicated that the *Fibrobacters* were significantly less (*p* = 0.001) active in the metatranscriptome as compared to the metagenome (Supplementary file S1).

**Figure 4:**
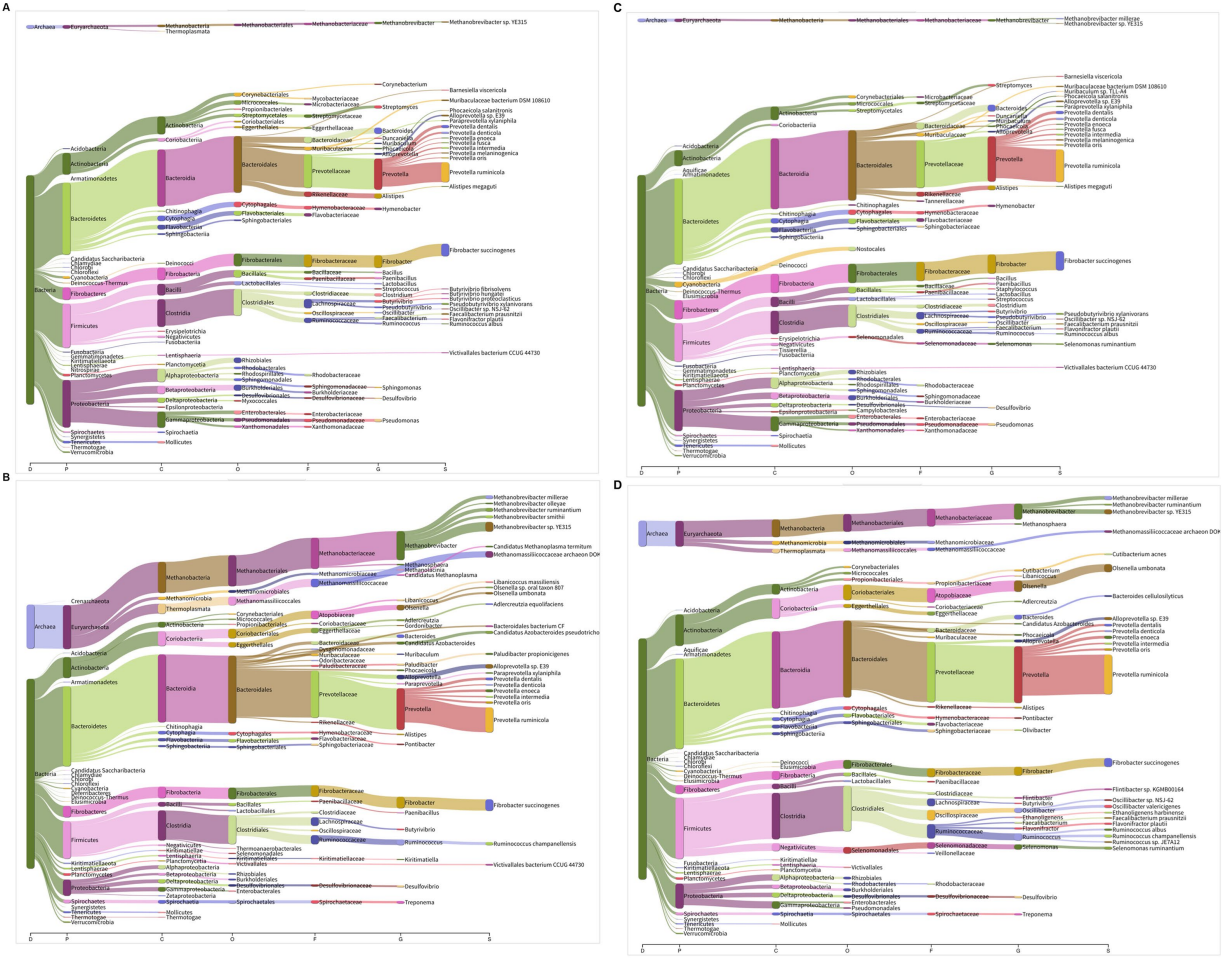
**(A–D)** Comparison of microbiota composition and functionality using Sankey diagram: **(A)** cattle metagenome, **(B)** cattle metatranscriptome, **(C)** buffalo metagenome, and **(D)** buffalo metatranscriptome. The branch width in Sankey diagram is proportional to microbial abundances or functionality in metagenome and metatranscriptome, respectively. Different letters at the x axis in Sankey diagram represent taxonomic ranks. D, domain; P, phylum; C, class; O, order; F, family; G, genus; S, species. The diagram depicts the representative metagenomes and metatranscriptomes profile of the rumen microbiota.

A representative visualization of the metagenome and metatranscriptome depicting the composition and functionality of the microbiota at various taxonomic ranks is presented in Figures 4A–D. Our results from the metatranscriptomics revealed that the archaea were functionally very active as compared to the compositional abundance (~15 vs. 1.5%). Methanogens from the phylum *Euryarchaeota* were more active in the rumen metatranscriptome than they were in the metagenome. The functionality of methanogens from the phylum *Crenarchaeota* was similar between the metagenome and transcriptome (Supplementary file S1), whereas the *Thaumarchaeota* phylum was not functional in the rumen metatranscriptome in both host species. In *Euryarchaeota*, the methanogens affiliated with the order *Methanobacteriales* were highly prominent and constituted 8–11% of the metatranscriptome, compared to 0.67–0.95% in the metagenome. Similarly, the *Methanomassiliicoccales*, *Methanosarcinales,* and *Methanomicrobiales* were also found to be comparatively more functional in the rumen metatranscriptome. On the contrary, the functionality of *Thermoplasmatales* was not different between the metagenome and transcriptome. Overall comparison of the metagenome and metatranscriptome revealed a significantly greater functionality of *Methanobrevibacter*, *Methanomassiliicoccus*, *Methanosphaera* and other methanogens. However, the functionality abundance of few methanogens specifically *Methanosarcina*, *Methanopyrus*, and *Methanohalophilus* were not different from the compositional abundances (Supplementary file S1).

### CAZymes in rumen metagenome and metatranscriptome

**Figure 5:**
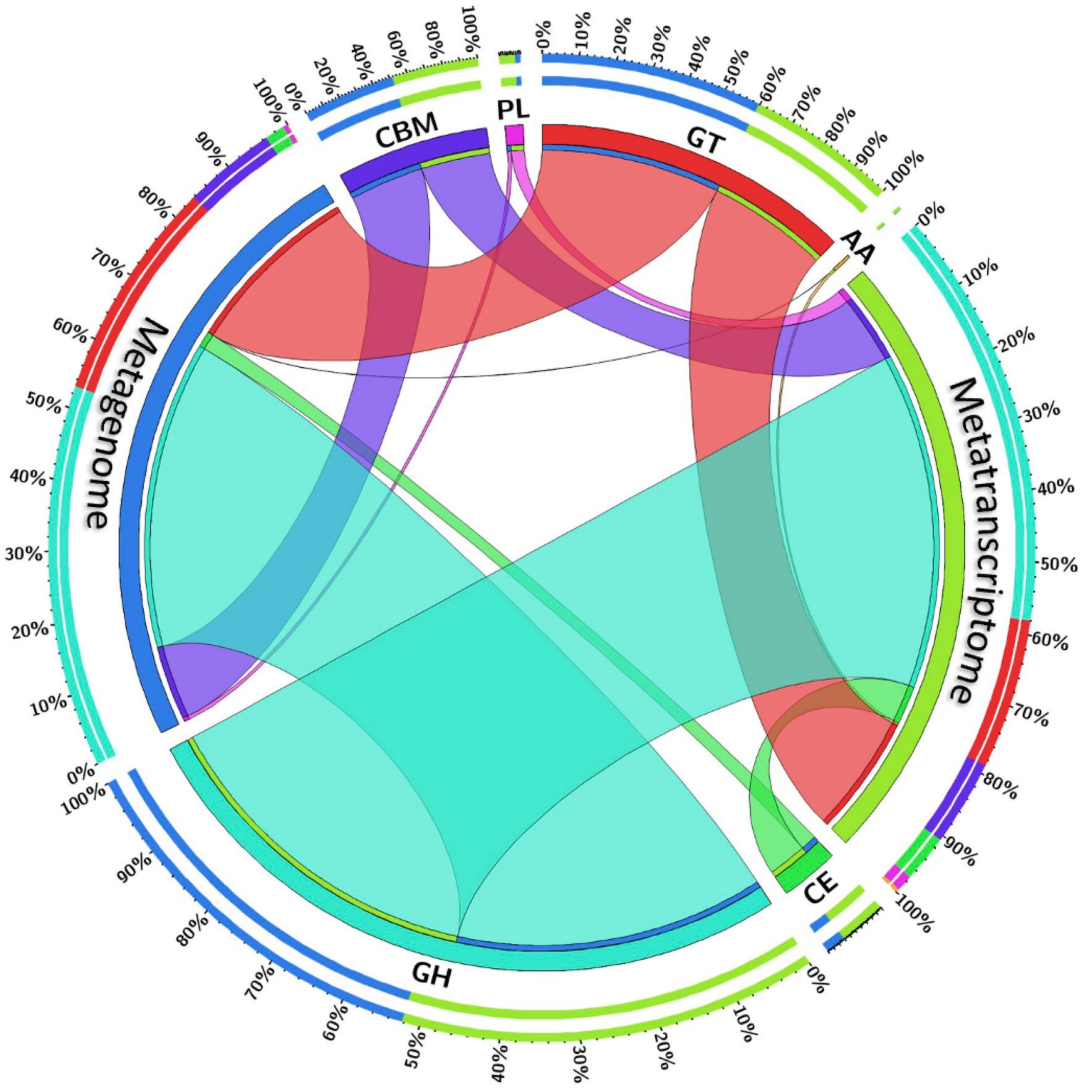
Chord diagram depicting the comparative functional capabilities of CAZymes in the rumen metagenome and metatranscriptome irrespective of the host species. Color of the chords represent CAZymes classes. The thickness of chord is proportional to CAZymes abundances in the metagenome and metatranscriptome. Outer circle depicts the percent abundance, whereas inner circle represents the CAZyme classes. MG, metagenome; MT, metatranscriptome; AA, auxiliary activities; CE, carbohydrate esterases; CBM, carbohydrate-binding modules; GH, glycoside hydrolases; GT, glycosyl transferases; PL, polysaccharide lyases.

Carbohydrate fermentation in the rumen is primarily driven by the microbial enzymes, and therefore the CAZymes belonging to different classes glycoside hydrolases, glycosyl transferases, carbohydrate binding molecules, carbohydrate esterases, polysaccharide lyases and auxiliary activities were screened in the rumen metagenome and metatranscriptome. The CAZymes profile of the rumen metagenome and metatranscriptome between cattle and buffaloes was compared to confirm if the functional capabilities between the two host species were different. Metagenome data were assembled into an average of 39,175 contigs per sample with an N50 average of 1,756 bp (Supplementary file S1), whereas metatranscriptome data were assembled into 13,295 contigs per sample (average N50 = 1,536 bp). A total of 159 and 133 CAZyme families were detected in the metagenome and metatranscriptome, respectively (Supplementary file S1). The contigs aligned against the CAZyme database revealed the dominance of glycoside hydrolases in both the metagenome (56–59%, Table 1) and metatranscriptome (52–53%), followed by glycosyl transferases, carbohydrate binding molecules and carbohydrate esterases. The CAZyme belonging to auxiliary activities was least abundant in both the metagenome and metatranscriptome (Table 1). Results from this study indicated that the CAZymes profile between cattle and buffaloes was similar in both the metagenome and metatranscriptome. However, a comparison of CAZymes revealed the greater (*p* < 0.0001) functionality of glycosyl transferases in the metatranscriptome than the rumen metagenome (Figure 5). On the contrary, the functional capability of the major CAZyme class glycoside hydrolases was lesser (*p* < 0.0001) in the metatranscriptome. Similarly, the carbohydrate esterases functionality in the metatranscriptome was also lesser than that in the rumen metagenome. There was no difference in the functional capability of auxiliary activities and carbohydrate binding molecules between the metagenome and metatranscriptome.

**Table 1:** Functional capabilities of CAZymes in the rumen metagenome and metatranscriptome of cattle and buffaloes fed on a finger millet straw and para grass-based diet.

Class	Cattle	Buffaloes	*p*-value
**Metagenome**			
Auxiliary activities (AA)	0.58	0.31	0.665
Carbohydrate-binding modules (CBM)	12.3	11.5	0.568
Carbohydrate esterases (CE)	6.15	7.07	0.181
Glycoside hydrolases (GH)	59.9	56.3	0.188
Glycosyl transferases (GT)	19.1	22.4	0.069
Polysaccharide lyases (PL)	2.01	2.47	0.326
**Metatranscriptome**			
Auxiliary activities (AA)	0.055	0.00	0.150
Carbohydrate-binding modules (CBM)	12.9	12.6	0.864
Carbohydrate esterases (CE)	3.02	2.51	0.440
Glycoside hydrolases (GH)	53.3	52.8	0.590
Glycosyl transferases (GT)	29.9	31.6	0.217
Polysaccharide lyases (PL)	0.812	0.443	0.177

### CH_4_ yield and rumen protozoa

Results from the study indicated higher (*p* = 0.003) daily enteric CH_4_ emissions in cattle than in buffaloes (Table 2). However, the correction of intake data revealed similar (*p* = 0.807) CH_4_ yield (g/kg DMI) between two host species. Similarly, results also indicated a similar enteric CH_4_ emission between cattle and buffaloes when compared per unit of digestible dry matter intake (45.4 vs. 46.5 g/kg DDM). The number of total protozoa (×10^7^ cells/ml) and Entodiniomorphs (*p* = 0.001) was significantly higher in buffaloes than in cattle, but the number of Holotrichs was the same in both hosts (Table 2).

**Table 2:** Daily enteric CH_4_ emissions, CH_4_ yield and protozoal population in cattle and buffaloes fed on a finger millet straw and para grass-based diet.

Attributes	Cattle	Buffaloes	*p*
Daily enteric CH_4_ (g/d)	174	130	0.003
CH_4_ yield (g/kg DMI)	26.9	26.7	0.807
CH_4_ emission (g/kg DDMI)	45.4	46.5	0.780
Total protozoa (×10^7^ cells/ml)	3.54	4.80	0.002
*Entodiniomorphs* (×10^7^ cells/ml)	3.46	4.72	0.001
*Holotrichs* (×10^7^ cells/ml)	0.085	0.079	0.852

DMI, dry matter intake; g/d, gram per day; DDMI, digestible dry matter intake. CH_4_, methane; g/d, gram per day; g/kg DMI, gram per kg of dry matter intake; P, probability of significance.

### Nutrient intake and digestibility

The digestibility study exhibited a higher (*p* < 0.0001) intake of DM, organic matter (OM), CP, and fiber fractions NDF and ADF in cattle than in buffaloes (Table 3). However, the nutrient digestibility was not different (*p* > 0.05) between the hosts.

**Table 3:** Nutrient intake and digestibility in cattle and buffaloes fed on a finger millet straw and para grass-based diet.

Attribute	Cattle	Buffaloes	*p*
**Intake**			
DM (kg/d)	6.58	4.95	<0.0001
OM (kg/d)	6.05	4.58	<0.0001
CP (g/d)	469	331	<0.0001
NDF (kg/d)	4.54	3.60	<0.0001
ADF (kg/d)	3.38	2.53	<0.0001
**Apparent digestibility (%)**			
DM	58.1	56.7	0.492
OM	60.2	58.4	0.381
CP	51.2	54.4	0.107
NDF	61.9	62.4	0.771
ADF	61.0	58.1	0.109

g/d, gram per day; kg/d, kilogram per day. Basal diet comprised finger millet straw and para grass in 70:30 ratio. The roughage diet contained 921 g OM, 56.1 g crude protein, 735 g NDF, 507 g ADF and 79.2 g ash per kg of DM. DM, dry matter; OM, organic matter; CP, crude protein; NDF, neutral detergent fiber; ADF, acid detergent fiber; P, probability of significance.

### VFA and ammonia

The fermentation parameters, such as total volatile fatty acid production (mmol) and ammonia-N (mg/L), were also similar (*p* > 0.05) between cattle and buffaloes. The concentration of individual fatty acids (mmol) except valerate also did not vary between the two host species (Table 4). The valerate production (mmol) in cattle was significantly higher (*p* = 0.035) than that in buffaloes. The acetate-propionate ratio, an indicator of the shift in fermentation, was also similar (4.84 vs. 4.88) between two bovine species.

**Table 4:** Comparative ruminal fermentation and volatile fatty acids profile in cattle and buffaloes fed on a finger millet straw and para grass-based diet.

Attribute	Cattle	Buffaloes	*p*
TVFA (mmol)	64.7	72.5	0.251
Ammonia (mg/L)	53.7	47.8	0.309
**Individual VFA**			
Acetate (mmol)	49.0	54.8	0.266
Propionate (mmol)	10.1	11.5	0.275
Butyrate (mmol)	4.38	5.24	0.123
Iso-butyrate (mmol)	0.457	0.341	0.135
Valerate (mmol)	0.467	0.320	0.035
Iso-valerate (mmol)	0.327	0.305	0.799
A/P ratio	4.88	4.84	0.845

TVFA, total volatile fatty acid; mmol, milli mole; mg/L, milligram per liter; A/P, acetate to propionate ratio; mmol, milli mole; P, probability of significance.

## Discussion

Rumen is considered a black box (Liu et al., 2021) consisting of diverse microbiota, including bacteria, archaea, protozoa, fungi, and viruses (Firkins and Yu, 2015), and perform complex functions in a syntrophic manner (Malik et al., 2022b). A consortium of rumen microbes from fermentation of feed produce volatile fatty acids (Ungerfeld, 2020) and microbial protein (Wallace et al., 1997), while methanogens co-exist with other rumen microbes and perform a different role in the scavenging of H_2_ (Morgavi et al., 2010). Thus, the rumen microbiota, due to its multifarious activities is considered equivalent to an organ (Huttenhower et al., 2012). Bacteria occupies the major niche in the microbiota and aids in anaerobic fermentation by the secretion of various enzymes (Li et al., 2017). Rumen microbiota composition is being affected by various factors such as diet (Liu et al., 2017), environmental conditions (Liu et al., 2021), and host species (Henderson et al., 2015; Roehe et al., 2016). Few studies reported the difference in rumen microbiota composition between the host species (Paul et al., 2017; Iqbal et al., 2018; Newbold and Ramos-Morales, 2020) and breed (Paz et al., 2016) fed on the same diet. On the contrary, some of the recent reports revealed similar microbiota compositions between the species (Henderson et al., 2015; Malik et al., 2021). Until now, many studies focusing on the rumen metagenome for revealing the microbial community composition in different species have been performed (Hess et al., 2011; Denman et al., 2015; Wallace et al., 2015; Delgado et al., 2019; Stewart et al., 2019; Gharechahi et al., 2020; Malik et al., 2021, 2022b); however, the studies investigating the metabolically active rumen microbiome through metatranscriptomics are relatively few (Li and Guan, 2017; Neves et al., 2020). Further, there is a dearth of reports investigating the structural composition of the rumen microbiota and their functional activities (Kamke et al., 2016; Li et al., 2019).

In this study, we have reported the microbiota affiliated with 41 phyla, 169 orders, 374 families, and 1,376 genera, which is consistent with the previous studies in cattle (Delgado et al., 2019; Wang et al., 2019; Kibegwa et al., 2023) and buffaloes (Sun et al., 2021; Li et al., 2022). *Bacteroidetes* and *Firmicutes* were the two most dominant bacterial phyla in both host species and constituted 53–58% of the metagenome. The dominance of *Bacteroidetes* and *Firmicutes* in the rumen microbiota is in good agreement with the previous reports (Jami and Mizrahi, 2012; Kim et al., 2014; Li et al., 2017; Söllinger et al., 2018). *Bacteroidetes* and *Firmicute*s are two major bacterial phyla that degrade complex plant polysaccharides and are involved in VFA production. *Bacteroidetes* possess a strong ability to degrade protein and polysaccharides (Huo et al., 2014; Pitta et al., 2016) and are considered major H_2_ utilizers in the rumen (Stewart et al., 1997). On the other hand, *Firmicutes* are known for their more H_2_ producing capabilities (Stewart et al., 1997). *Firmicutes* are efficient in breaking lignocellulosic complexes, and therefore their high abundance may increase carbohydrate fermentation in the rumen (Fernando et al., 2010). The F/B ratio (0.55–0.77) in the metagenome in our study is consistent with the previous findings of Malik et al. (2022a) Fernando et al. (2010), and Nathani et al. (2015), and the negative correlation (*r* = −0.81) between *Firmicutes* and *Bacteroidetes* is corroborated with the previous reports (Li et al., 2019; Malik et al., 2022b). *Bacteroidetes* and *Firmicutes* due to their involvement in the degradation of complex plant polysaccharides, breaking of lignocellulosic complexes, H_2_ production, and utilization capabilities, are prominent in the rumen microbiota. A non-significant difference in the apparent digestibility of fiber fractions, i.e., NDF, ADF, and CP, can be attributed to the comparable distribution of *Bacteroidetes* (*p* = 0.355) and *Firmicutes* (*p* = 0.416) in the rumen metagenome of cattle and buffaloes fed on the high-fiber diet consisting of finger millet straw and para grass in 70:30. Moreover, the similar VFA production in both host species did not imply any difference in the activities of the two prominent bacterial phyla. The similar activities of the *Bacteroidetes* and *Firmicutes* can also be established by the similar metagenome CAZymes profiles between the two host species. The similar distribution of *Bacteroidetes* and *Firmicutes* phyla in cattle and buffaloes was consistent with the previous findings of Sun et al. (2021). On the contrary, Iqbal et al. (2018) and Wang et al. (2020) reported higher distributions of *Bacteroidetes* in cattle and *Firmicutes* in buffaloes. The disagreement in the distribution of *Bacteroidetes* and *Firmicutes* in our study with the previous findings (Iqbal et al., 2018; Wang et al., 2020) could be attributed to diet composition, which has a remarkable impact on rumen microbiota. *Firmicutes* distribution tends to increase with the increasing roughage proportion in diet (Terry et al., 2019). Thus, it may be inferred that the distribution of *Bacteroidetes* and *Firmicutes* remains similar between cattle and buffaloes on a roughage-based diet, while concentrate feeding may lead to a differential distribution of the above two phyla between cattle and buffaloes.

Despite the sole roughage based diet in present study, the *Proteobacteria* constituted the third most abundant bacterial phylum in both the cattle and buffaloes and the difference between two host species was not significant. The deviation in the *Proteobacteria* abundance from the earlier reports could be attributed to the variation in diet composition (Hu et al., 2020; Kibegwa et al., 2023).

In this study, the methanogens affiliated to the phylum *Euryarchaeota*, *Crenarchaeoeta*, *Thaumacrhaeota* and *Geothermarchaeota* were identified. The overall abundance of archaea in the rumen microbiota was in good agreement with the earlier reports (Janssen and Kirs, 2008; Comtet-Marre et al., 2017; Zhu et al., 2017; Malik et al., 2022b). Among the archaeal phylum, *Euryarchaeota* was the most abundant, constituted 1.10-1.48% of the total rumen microbiota. Earlier studies also reported the dominance of *Euryarchaeota* in the archaeal phyla (Malik et al., 2021, 2022a, 2022b). The abundance of archaeal phyla were comparable between cattle and buffaloes. *Methanobrevibacter,* with a mean abundance of 0.7–1.0% of the rumen metagenome, was the most prominent genus of methanogens in both cattle and buffaloes, and their abundance was comparable between the two host species. In a previous study, Malik et al. (2021) also reported the dominance and similar abundance of *Methanobrevibacter* in the rumen metagenome of cattle and buffaloes fed on hybrid Napier and concentrate-based diet. The dominance of *Methanobrevibacter* among the rumen archaea is in congruence with the earlier reports in cattle (Sirohi et al., 2013; Danielsson et al., 2017; Parmar et al., 2017; Pitta et al., 2021) and buffaloes (Franzolin et al., 2012; Kumar et al., 2018). A diverse community of hydrogenogenic fermenters and hydrogenotrophic methanogens, along with other reducing microbes, co-exists in the rumen (Greening et al., 2019). Fermentation of fibrous feed in the rumen is associated with H_2_ production, which is primarily taken up by the methanogens (Lathamt and Wolintt, 1977; Morgavi et al., 2010), and that is why the hydrogenotrophic pathway is most prominent in the rumen (Hungate, 1967; Friedman et al., 2017). The dominance of *Methanobrevibacter* in the rumen archaeal community could be explained by its association with H_2_ production and subsequent reduction of CO_2_ to CH_4_ through the Wolfe cycle (Leahy et al., 2010; Thauer, 2012).

Methanogens such as *Methanococcales*, *Methanomassiliicoccales*, *Methanomicrobiales*, *Methanosarcinale*s, *Methylococcales,* and *Thermoplasmatales* together constituted a minor fraction (0.37–0.50%) of the rumen metagenome in cattle and buffaloes. The aggregate abundance (%) of other methanogens in the rumen of cattle and buffaloes is consistent with Morgavi et al. (2010), who reported that the methanogens other than *Methanobrevibacter* constituted nearly one-third of the rumen archaea. Though the hydrogenotrophic pathway is most prominent in methanogenesis (Pitta et al., 2021), aceticlastic and methylotrophic pathways also make small contributions to rumen methanogenesis (Janssen and Kirs, 2008; Liu and Whitman, 2008; Morgavi et al., 2010). Overall, hydrogenotrophs affiliated with the *Methanobrevibacter* genus are the most abundant methanogens in the rumen of cattle and buffaloes, and our results are substantiated by the findings of Martínez-Álvaro et al. (2020), who reported that the hydrogenotrophic functional niche is different from that of methylotrophic methanogens. Substrate combined with the thermodynamics of methanogenic pathways (hydrogenotrophic > Methylotrophic > Aceticlastic) could explain the higher abundance of hydrogenotrophic over aceticlastic and methylotrophic methanogens in both cattle and buffaloes. It is hypothesized that the high concentration of H_2_ in the rumen exceeds the threshold of hydrogenotrophs due to the abundant availability of CO_2_ and outcompetes the methylotrophic (Pitta et al., 2021) and aceticlastic methanogens.

The significantly higher enteric CH_4_ emissions (g/d) in cattle can be attributed to the comparatively higher body weight and feed intake than the buffaloes. However, the CH_4_ yield (g/kg DMI) was comparable between the two host species fed on analogue diet comprising finger millet straw and para grass. Apart from the archaeal community, fermentation products such as VFA also affect enteric methanogenesis. VFA are mid products of OM fermentation, and their relative proportions affect the extent of ruminal methanogenesis (Dijkstra, 1994; Malik et al., 2017). The conversion of VFAs to CH_4_ varies considerably in the following order: acetate > butyrate > propionate (Wang et al., 2009). Earlier studies concluded that the accumulation of propionate inhibits the activity of methanogens and therefore leads to a reduction in CH_4_ emissions (Yeole et al., 1997; Demirel and Yenigün, 2002). Since the concentrations of acetate, propionate, and butyrate were similar, the comparable CH_4_ yield in both host species can be aggregately attributed to the non-significant difference in VFA production and microbiota. These findings are in consonance with our previous study, where the VFA profile and CH_4_ yield were comparable between cattle and buffaloes fed a hybrid Napier and concentrate-based diet in 70:30 (Malik et al., 2021). Similarly, Iqbal et al. (2018) also reported similar acetate and propionate production between cattle and buffaloes when fed on an analogue diet consisting of concentrate and corn silage.

Protozoa, after bacteria, are the second most abundant microbes in the rumen and constitute about half of the rumen biomass (Newbold et al., 2015). Protozoa are involved in the interspecies H_2_ transfer to the methanogenic archaea (Li et al., 2018), and therefore a reduction in rumen protozoal numbers may lead to an indirect inhibition of methanogenesis (Bhatta et al., 2009; Gemeda and Hassen, 2015; Malik et al., 2017; Thirumalaisamy et al., 2022a). About 37% of rumen methanogenesis is associated with protozoa (Finlay et al., 1994; Machmüller et al., 2003), and CH_4_ emissions are linearly linked with the protozoal population; however, there are also protozoa-independent mechanisms that regulate methanogenesis (Guyader et al., 2014). Despite a significant difference in protozoa numbers, the similar CH_4_ yield in host species could be due to the comparable fermentation, VFA profile, and microbiota that aggregately coupled with the H_2_ production as well as utilization. Recently, Dai et al. (2022), in a meta-analysis, concluded that the simple illustration of rumen protozoa without looking at the category could not explain the impact on methanogenesis. It has been established that isotrichids (holotrichs) rather than entodiniomorphs have a more pronounced effect on methanogenesis (Belanche et al., 2015; Dai et al., 2022). These findings also strengthen our data that, despite the different numbers of protozoa and entidiniomorphs in cattle and buffaloes, the comparable isotrichids (holotrichs) are attributed to the similar CH_4_ yield.

Metatranscriptome data also established that, apart from their dominance in the rumen microbiota, the *Bacteroidetes* and *Firmicutes* were functionally the two most active microbial phyla in both cattle and buffaloes. At the genus level, fibrolytic bacteria such as *Prevotella*, *Fibrobacter,* and *Ruminococcus* were functionally most active in the rumen microbiota. All together, these three genera constituted more than 1/3rd of the metatranscriptome; however, there was no difference in the functionality of these microbes between cattle and buffaloes. Since there was no difference in the CAZymes profile in the metatranscriptome between the cattle and buffaloes, the apparent digestibility of nutrients was similar among the host species. Glycoside hydrolase (GH) is one of the crucial categories of enzymes accountable for carbohydrate degradation in the rumen by loosening cellulose surfaces, peeling the fibers, and pushing the cellulose chain into the catalytic core for conversion to substrates (Kataeva et al., 2002). GH enzymes hydrolyze the glycosidic bonds between the sugars or a sugar and a non-sugar moiety (Lehninger, 2005). Cellulases are complex enzymes consisting of consortia of enzymes such as endo-β-1,4-glucanases, cellobiohydrolases, cellodextrinases, and β-glucosidases (Sathya and Khan, 2014). In this study, the cellulase type was represented by 15 families, mainly encoding endoglucanases (8 out of 31 families). The GH families identified in the metatranscriptome were consistent with the previous reports (Seshadri et al., 2018; He et al., 2019; Wang et al., 2019; Neves et al., 2021). A comparison of GH CAZymes revealed that the functionality of GH5, GH8, GH16, GH51, GH30, and GH95 was significantly less in the metatranscriptome than in the metagenome. Moreover, there were some GH families (GH45, GH74, and GH116) that remained undetected in the metatranscriptome. The degradation of cellulose and hemicellulose is carried out by a synergistic action of GH CAZymes (Terry et al., 2021), and the less active or absence of several GHs in the rumen proteome like in this study has been reported previously (Neves et al., 2021). On the other hand, GH48 was functionally more active in the transcriptome than the metagenome. Undisputedly, genes encoding for fiber degradation usually constitute major fractions of the functional microbes in the metatranscriptome. Earlier studies (Dai et al., 2015; Comtet-Marre et al., 2017) support our findings for higher functionality of GH48 in the metatranscriptome. Our results established that suboptimal expression of many CAZymes families in the metatranscriptome does not affect fiber degradation, as evidenced by the similar NDF and ADF digestibility, because fiber degradation in the rumen is carried out by a consortia of rumen microbes, specifically bacteria, in a synergistic fashion (Koike and Kobayasi, 2009; Ribeiro et al., 2016). The suboptimal expression of CAZymes in the metatranscriptome has also been reported in previous studies (Hess et al., 2011; Li et al., 2019).

Methanogens from the phylum *Euryarchaeota* were relatively more active in the rumen metatranscriptome than the metagenome; however, the difference in the functionality of *Euryarchaeota* between cattle and buffaloes was not significant, and that is why the CH_4_ yield (g/kg DMI) was comparable between the host species. The similar CH_4_ yield between the host species can be attributed to the analogue microbiota profile and diet composition (Malik et al., 2015a), and the host species appears to have minimal control over the rumen methanogenesis under uniform environmental conditions (Malik et al., 2021). The CH_4_ yield in cattle and buffaloes in this study was in good agreement with the previous reports and global datasets (Hristov et al., 2013; Pinares-Patino et al., 2014; Charmley et al., 2016; Malik et al., 2021, 2022a).

Despite the restricted depiction (Yanagita et al., 2000; Henderson et al., 2015) in the rumen metagenome (~1.5%), archaea perform a specialized function of scavenging metabolic H_2_ via reducing CO_2_ into CH_4_, which is why their functionality in the metatranscriptome was quite high (~15%) as compared to the metagenome. Three pathways, namely hydrogenotrophic, methylotrophic, and aceticlastic, of methanogenesis exist in the rumen; however, among the three, CH_4_ production via the hydrogenotrophic pathway is the most prominent in the rumen (Hungate, 1966; Hungate et al., 1970; Friedman et al., 2017; Malik et al., 2022a). Hydrogenotrophic methanogens belong to the archaeal largest phylum, *Euryarchaeota,* in the rumen (Kim et al., 2011; Malik et al., 2022a). Though the contribution of the *Crenarchaeota* and *Thaumarchaeota* phyla (Shin et al., 2004; Abecia et al., 2014; Xue et al., 2019) to the rumen methanogenesis is not yet determined, based on their abundances, it may be hypothesized that these phyla may not contribute significantly to the rumen methanogenesis. In agreement with the previous studies (Wright et al., 2004; Seedorf et al., 2015; Huang and Li, 2018; Malik et al., 2021, 2022a,b), *Methanobacteriales was the* most prominent order of the methanogens in the present study, and the metabolic functionality of the methanogens affiliated to this order was quite high in the metatranscriptome as compared to the metagenome (8–11 vs. 0.67–0.95%). Similarly, the *Methanomassiliicoccales and Methanomicrobiales* functionality in the metatranscriptome was also significantly higher than that in the metagenome. However, the metabolic functionality of the methanogens from these three orders in the rumen metatranscriptome between cattle and buffaloes was comparable. However, *Thermoplasmatales* compositional abundances (metagenome) and metabolic functionality (metatranscriptome) were similar, which indicates that they may not be playing an important role in rumen methanogenesis. Among the hydrogenotrophic methanogens, *Methanobrevibacter* alone constituted about 10% of the metabolically active metatranscriptome, which is far ahead of their compositional representation (0.85%) in the metagenome. The dominance of *Methanobrevibacter* in the archaeal community is consistent with the previous reports (Janssen and Kirs, 2008; Snelling et al., 2014; Seedorf et al., 2015; Danielsson et al., 2017; Malik et al., 2021).

## Conclusion

The results indicate that the bacterial community in the rumen was more diverse than the archaeal community. *Bacteroidetes* and *Firmicutes* were prominent bacterial phyla in the constitution of the metagenome and the metabolic functionality of the metatranscriptome of cattle and buffaloes fed a similar diet of finger millet straw and para grass. There was no distinction between the compositional (metagenome) and functional (metatranscriptome) abundances of these two phyla. In contrast, despite their small proportion in the metagenome, archaea comprise nearly 15% of the most functionally active components of the metatranscriptome. *Euryarchaeota* was the most numerous and functionally active phylum of methanogens, while *Methanobacteriale*s and *Methanobrevibacter* were the most prevalent order and genus of rumen methanogens in cattle and buffaloes. The study revealed substantial variation in the compositional abundances and metabolic functionality of the rumen metagenome and metatranscriptome, respectively; however, the compositional structure and metabolic functionality of the rumen microbiota of cattle and buffaloes were comparable. Similar microbiota composition and metabolic functionality in both host species result in comparable CH_4_ production. The relatively greater metabolic functionality of methanogens compared to their metagenomic representation suggests that they serve a specialized function in the rumen. In contrast, the lower/higher activities of some CAZymes in the metatranscriptome indicate that fiber digestion is a function performed by a consortium of microorganisms, as evidenced by the similar fiber digestibility of cattle and buffaloes. Overall, the study determined that feeding cattle and buffaloes the same diet resulted in similar microbiota composition and metabolic functionality, resulting in comparable CH_4_ production. The effect of varying diets and environments on the composition and consequent metabolic functionality of the rumen microbiota, as well as their effect on CH_4_ production, requires additional study.

## Data availability statement

The datasets presented in this study can be found in online repositories. The metagenome data is deposited in the NCBI database and can be found at: https://www.ncbi.nlm.nih.gov/bioproject/PRJNA949096. Metatranscriptomics data can be accessed at: https://www.ncbi.nlm.nih.gov/bioproject/PRJNA949119.

## Ethics statement

The animal study was approved by Committee for Control and Supervision of Experiments on Animals (CPCSEA), Ministry of Fisheries, Animal Husbandry, and Dairying, Government of India (Approval no. NIANP/IAEC/1/2020/5). The study was conducted in accordance with the local legislation and institutional requirements.

## Author contributions

PM: Conceptualization, Project administration, Resources, Supervision, Writing – original draft, Writing – review & editing. ST: Investigation, Methodology, Visualization, Writing – original draft. AK: Investigation, Methodology, Software, Visualization, Writing – original draft, Formal analysis. AM: Investigation, Methodology, Software, Visualization, Writing – original draft. SB: Investigation, Methodology, Writing – original draft. AB: Investigation, Methodology, Writing – original draft. RB: Funding acquisition, Project administration, Resources, Supervision, Writing – review & editing. HR: Funding acquisition, Project administration, Resources, Supervision, Writing – original draft.
